# Potentiation of Anticancer Drugs: Effects of Pentoxifylline on Neoplastic Cells

**DOI:** 10.3390/ijms13010369

**Published:** 2011-12-28

**Authors:** Miroslav Barancik, Viera Bohacova, Lenka Gibalova, Jan Sedlak, Zdena Sulova, Albert Breier

**Affiliations:** 1 Institute for Heart Research, Slovak Academy of Sciences, Dúbravská cesta 9, Bratislava 840 05, Slovakia; 2 Institute of Molecular Physiology and Genetics, Centre of Excellence of the Slovak Research and Development Agency “BIOMEMBRANES 2008” Slovak Academy of Sciences, Vlárska 5, Bratislava 83334, Slovakia; E-Mails: viera.bohacova@savba.sk (V.B.); lenka.gibalova@savba.sk (L.G.); zdena.sulova@savba.sk (Z.S.); 3 Cancer Research Institute, Slovak Academy of Sciences, Vlárska 7, Bratislava 833 91, Slovakia; E-Mail: jan.sedlak@savba.sk

**Keywords:** pentoxifylline, P-glycoprotein, multidrug resistance, apoptosis, matrix metalloproteinases, caspases

## Abstract

The drug efflux activity of P-glycoprotein (P-gp, a product of the *mdr1* gene, ABCB1 member of ABC transporter family) represents a mechanism by which tumor cells escape death induced by chemotherapeutics. In this study, we investigated the mechanisms involved in the effects of pentoxifylline (PTX) on P-gp-mediated multidrug resistance (MDR) in mouse leukemia L1210/VCR cells. Parental sensitive mouse leukemia cells L1210, and multidrug-resistant cells, L1210/VCR, which are characterized by the overexpression of P-gp, were used as experimental models. The cells were exposed to 100 μmol/L PTX in the presence or absence of 1.2 μmol/L vincristine (VCR). Western blot analysis indicated a downregulation of P-gp protein expression when multidrug-resistant L1210/VCR cells were exposed to PTX. The effects of PTX on the sensitization of L1210/VCR cells to VCR correlate with the stimulation of apoptosis detected by Annexin V/propidium iodide apoptosis necrosis kit and proteolytic activation of both caspase-3 and caspase-9 monitored by Western blot analysis. Higher release of matrix metalloproteinases (MMPs), especially MMP-2, which could be attenuated by PTX, was found in L1210/VCR than in L1210 cells by gelatin zymography in electrophoretic gel. Exposure of resistant cells to PTX increased the content of phosphorylated Akt kinase. In contrast, the presence of VCR eliminated the effects of PTX on Akt kinase phosphorylation. Taken together, we conclude that PTX induces the sensitization of multidrug-resistant cells to VCR via downregulation of P-gp, stimulation of apoptosis and reduction of MMPs released from drug-resistant L1210/VCR cells. These facts bring new insights into the mechanisms of PTX action on cancer cells.

## 1. Introduction

A major problem in cancer chemotherapy is the development of a multidrug resistance (MDR) phenotype in cancer cells. The drug efflux activity of P-glycoprotein (P-gp, a product of the *mdr1* gene and an ABCB1 member of the ABC transporter family) represents a mechanism by which tumor cells escape death induced by chemotherapeutic agents, resulting in the development of MDR in cancer cells and causing them to become insensitive to a range of different cytostatic drugs [1,2]. Pentoxifylline (PTX) is a methylxanthine well-known for its roles as a phosphodiesterase inhibitor, a ligand of adenosine receptors, a modulator of the ryanodine Ca^2+^-release channel of the sarco(endo)plasmic reticulum, and a downregulator of TNF-α [3–6]. Additional reports also suggest that PTX administration might increase the effectiveness of antitumor chemotherapy [7,8], and our previous studies demonstrated that PTX induced the reversal of vincristine (VCR) and/or adriamycin resistance in P-gp-positive L1210/VCR (P-gp positive cell variant of L1210 cells were obtained by selection with VCR) mouse leukemia cells [9,10]. However, the mechanism of PTX action on drug resistance of cancer cells still remains unclear.

P-gp-positive L1210/VCR mouse leukemia cells were found to be defective in drug-induced apoptosis, even for drugs that are not substrates of P-gp [11]. Recently, sensitization of cervical cancer cells to adriamycin by PTX was found to be associated with more pronounced apoptosis [12]. Here, we tested the hypothesis that PTX restores the sensitivity of L1210/VCR cells to VCR due to a decrease in P-gp protein and that this effect is associated with a stimulation of apoptosis. We described the MDR reversal effect of LY294,006, (an inhibitor of PI3K/Akt kinase pathway) in L1210/VCR cells [13]. Pentoxyfilline was described to diminish activation of Akt kinase induced by chronic common bile duct ligation lung [14]. Therefore, we tested the hypothesis if PTX effect on MDR reversal in L1210/VCR cells is associated with modulation of Akt kinase levels or activation. PTX was also found to inhibit the proliferation of B16F10 melanoma cells by inhibiting cell adhesion and secretion of matrix metalloproteinases (MMPs) [15]. Another study reported that lung carcinoma cell variants obtained by selection with anticancer drugs showed enhanced invasive abilities through the upregulation of MMPs [16]. In order to determine the role of MMPs in the PTX-induced effects on drug-resistant L1210/VCR cells, we examined the influence of PTX on these enzymes. Current paper is aiming to study if exist any relation between PTX induced P-gp downregulation in L1210/VCR cells and the effect of PTX on: (i) apoptosis induced by vincristine detected by annexin V binding and caspases activation; (ii) Akt kinase levels and activation and (iii) MMPs release.

## 2. Results and Discussion

### 2.1. Results

#### 2.1.1. Pentoxifylline Reduces P-glycoprotein Protein Levels

**Figure 1:**
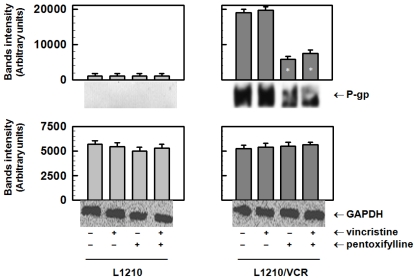
Reduction of P-glycoprotein (P-gp) protein levels by pentoxifylline (PTX). P-gp protein levels were analyzed L1210 and L1210/VCR cells treated with 1.2 μmol/L vincristine (VCR) and/or 100 μmol/L PTX for 24 h. The protein levels of P-glycoprotein and GAPDH (housekeeper) were determined by Western blot analysis using specific antibodies. Blots of three independent experiments were quantified and obtained data were evaluated by Student’s *t* test. Data represent means ± SD. * Different from control (without drugs) at significance level *p* < 0.01.

P-gp in membrane fraction isolated from P-gp-positive L1210/VCR cells was detected by Western blot analysis using a c219 antibody (Figure 1).

The parental cells did not contain any detectable amount of P-gp under the applied experimental conditions. Cultivation of resistant cells in the presence of 1.2 μmol/L VCR induced only a slight (if any) increase in P-gp protein levels. VCR at this concentration induced massive cell damage in L1210 cells but did not influence the survival of L1210/VCR cells [17]. In contrast, PTX at a concentration of 100 μmol/L induced a strong decrease in P-gp protein levels in L1210/VCR cells. PTX alone at this concentration did not influence the viability of either L1210 or L1210/VCR cell lines but induced a massive reversal of VCR resistance in L1210/VCR cells [10,18,19].

#### 2.1.2. Drug Reversal Effects of Pentoxifylline Are Associated with the Stimulation of Apoptosis

**Figure 2:**
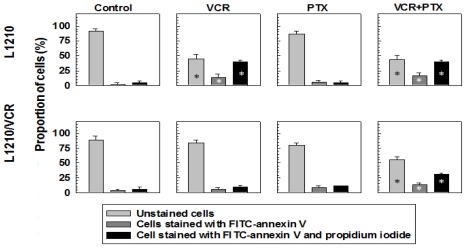
The effects of VCR and PTX on apoptosis induction in sensitive (L1210) and multidrug-resistant (L1210/VCR) cells. Cells were exposed to VCR (1.2 μmol/L) and/or PTX (100 μmol/L) for 24 h. Induction of apoptosis was detected by flow cytometry using an Annexin V-FITC Apoptos1is detection kit. Staining of cells by annexin V-FITC or propidium iodide was determined by measuring the fluorescence of cells using a Coulter Epics Altra flow cytometer. The proportions of cells stained with annexin V-FITC (early apoptotic cells), cells with both annexin V-FITC and propidium iodide (late apoptotic/necrotic cells) and unstained cells (normal cells) are summarized. Data represent means ± SD from free independent experiments. * Different from control (without drugs) at significance levels *p* < 0.01.

**Figure 3:**
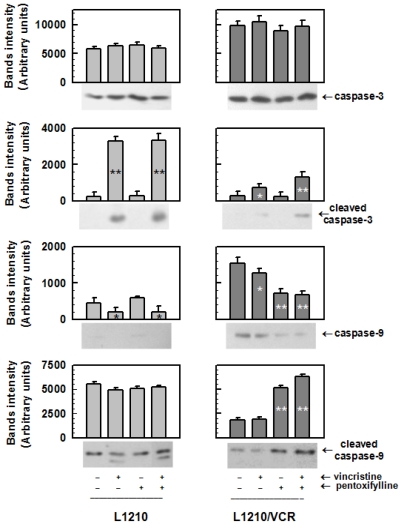
Effect of VCR and PTX treatment on caspase-3 and caspase-9. The sensitive L1210 and resistant L1210/VCR cells were treated with VCR (1.2 μmol/L) and/or PTX (100 μmol/L) for 24 h. The protein levels of full-length and cleaved caspase-3 and caspase-9 were determined by Western blot analysis using specific antibodies. Blots of three independent experiments were quantified and obtained data were evaluated by Student’s *t* test. * And ** different from control (without drugs) at significance level *p* < 0.01 and *p* < 0.05, respectively.

The presence of VCR alone in the cultivation medium induced a massive increase in the percentage of apoptotic cells in sensitive L1210 cells but not in resistant L1210/VCR cells (Figure 2). This could be detected as early (cells labelled with annexin V only) and late (cells labelled by both annexin V and propidium iodide) apoptosis and was determined by flow cytometry using the Annexin V-FITC Apoptosis detection kit. However, the presence of both VCR and PTX induced an increase in the number of early and late apoptotic L1210/VCR cells (Figure 2). This may be ascribed to a PTX-induced reversal of VCR resistance in L1210/VCR cells, as described previously [10,18,19], because PTX alone induced only slight changes in the apoptotic progression of L1210 or L1210/VCR cells. However, PTX alone induced an increase in cleaved/activated caspase-9 (an enzyme known to be active in apoptosis) (Figure 3) corresponding to a decrease in the non-cleaved form of this enzyme in P-gp-positive L1210/VCR cells. Activation of this enzyme under the influence of PTX was not observed in sensitive L1210 cells. When investigating the effects of PTX on caspase-3 (an enzyme downstream of caspase-9), we did not find significant differences in the levels of inactive zymogen in either sensitive L1210 or resistant L1210/VCR cells (Figure 3). VCR induced an increase in the levels of both cleaved caspase-3 and caspase-9 in sensitive cells. These effects of VCR on caspase activation were not observed in the resistant cell variant. However, combined treatment of P-gp-positive L1210/VCR cells with both PTX and VCR induced a more pronounced proteolytic activation of both caspase-3 and caspase-9.

#### 2.1.3. Pentoxifylline Influences the Activation of Akt Kinase in Resistant L1210/VCR Cells

**Figure 4:**
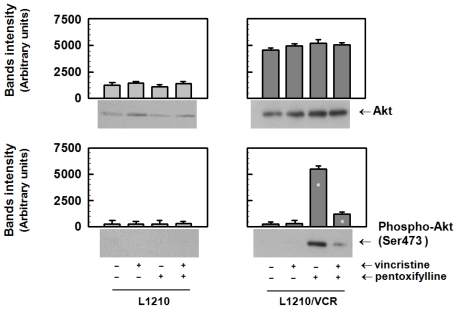
Effect of VCR and PTX treatment on Akt kinase protein expression and specific phosphorylation. The sensitive L1210 and resistant L1210/VCR cells were treated with 100 μmol/L PTX and/or 1.2 μmol/L VCR for 24 h, Akt kinase protein expression and its specific fosforylation (on Ser 473) were determined by Western blot analysis using a specific antibodies. Blots of three independent experiments were quantified and obtained data were evaluated by Student’s *t* test. * Different from control (without drugs) at significance level *p* < 0.01.

Resistant variant of L1210 cells contains higher content of Akt kinase protein than sensitive parental cells (Figure 4). The incubation of both sensitive L1210 and resistant L1210/VCR cells in the presence of VCR and/or PTX for 24 h did not influence the protein levels of Akt kinase (Figure 4). Akt kinase activity is stimulated via phosphorylation of Ser473 by upstream protein kinases [20]. Therefore, the amounts of Ser473-phosphorylated Akt kinase were investigated using a specifically reacting antibody. Sensitive and resistant cells did not contain any detectable Akt kinase phosphorylated on Ser473 at basal conditions or in the presence of VCR. In contrast to sensitive L1210 cells, exposure of resistant L1210/VCR cells to 100 μmol/L PTX for 24 h markedly increased the phosphorylation of Akt kinase (Figure 4).

However, presence of VCR during treatment of resistant cells with PTX induced depression of Akt kinase phosphorylation.

#### 2.1.4. Pentoxifylline Affects the Release of Matrix Metalloproteinases from L1210/VCR Cells

**Figure 5:**
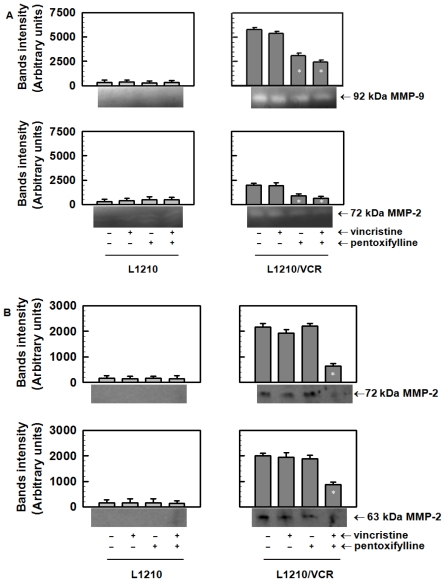
Effects of VCR and PTX treatment on the secretion of matrix metalloproteinases from sensitive L1210 and resistant L1210/VCR cells into the external medium. (**A**) Zymogram showing the activities of matrix metalloproteinases analyzed using gelatin zymography; (**B**) The protein expression of secreted MMP-2 was analyzed by Western blot analysis using a specific antibody that reacts with both the 72 and 63 kDa forms of MMP-2. Zymograms and blots of three independent experiments were quantified and obtained data were evaluated by Student’s *t* test. * Different from control (without drugs) at significance level *p* < 0.01.

The activities of matrix metalloproteinases released from sensitive L1210 and resistant L1210/VCR cells into external (conditioned) medium were analyzed by gelatin zymography. Recombinant, active MMP-2 was used as a positive control to identify the active 63 kDa MMP-2 form. Pro-MMP-2 and MMP-9 were identified using fetal bovine serum containing predominantly these forms of both MMPs. Gelatin zymography of the concentrated conditioned media revealed gelatinolytic activities corresponding to MMP-2 and MMP-9 in samples from L1210/VCR cells but not from L1210 cells (Figure 5A). This indicated an increased secretion of both enzymes from the P-gp-positive cell variant. The zymogram for L1210/VCR cells showed the presence of gelatinolytic activities at 92 kDa and 72 kDa, which represent the latent forms of both MMP-9 and MMP-2, respectively (Figure 5A). However, gelatinolytic activities were not detected at 88 and 63 kDa, which represent the cleaved forms of MMP-9 and MMP-2, respectively. The increased secretion of matrix metalloproteinases from L1210/VCR cells could be attenuated by PTX but not by VCR (Figure 5A).

We detected the presence of MMP-2 by Western blot analysis, using a specific antibody that recognized both the latent (72 kDa) and cleaved form (63 kDa) of MMP-2 in the conditioned medium obtained after incubation of L1210/VCR (Figure 5B). In contrast, conditioned medium obtained after incubation of L1210 cells did not contains detectable amounts of MMP2 forms (Figure 5B). Combine treatment of L1210/VCR cells by PTX and VCR decreased content of both forms of MMP-2.

### 2.2. Discussion

Previously, we have described a reduction in P-gp mRNA in L1210/VCR cells after PTX treatment [9,19]. This P-gp downregulation observed in L1210/VCR cells was assumed to be responsible for the reversal of the adriamycin or VCR resistance in this cell model [9,19] and the elevation of the intracellular concentrations of both cytostatics. The ability of PTX to potentiate adriamycin toxicity was also described in chronic human myeloid leukemia cells and in P388 leukemia cells [21,22]. In the current paper, we demonstrated a decrease in P-gp protein expression after PTX treatment (Figure 1). This effect of PTX on P-gp cellular expression could be achieved at a PTX concentration that did not induce significant cell damage to L1210 cells or its MDR variants [9,10,18,19].

Interestingly, in contrast to PTX, which induced a reversal of the P-gp mediated MDR in L1210/VCR cells [18], other xanthines, such as caffeine or theophylline, did not induce such an effect [10]. Moreover, caffeine was not able to alter *mdr1* mRNA levels [19]. The fact that both the caffeine and theophylline are effective in the generally known pharmacological roles of xanthines (described in the introduction) but not in the modulation of P-gp mediated MDR fails to satisfactorily explain the VCR-induced reversal of MDR on the basis of common pharmacological effects of xanthines. Neither PTX nor its major oxidative metabolites influenced the ATPase activity measured in membrane vesicles isolated from Sf9 insect cells infected with a baculovirus containing the respective genes for ABC transporters such as P-gp [23].

This suggests that PTX does not induce its effects on P-gp-mediated MDR via alteration of ATP hydrolysis. Instead, PTX was found to sensitize the multidrug-resistant mouse leukemia cell line L1210/VCR to VCR by stimulating apoptosis. Similarly to PTX, LY294,002 was also found to increase apoptosis induced by vincristine in this cell model [13]. However, in contrast to PTX that induces an elevation of Akt kinase activation (Figure 4) LY294,002, as PI3K/Akt kinase pathway inhibitor, induced opposite effect. Akt kinase is believed to play an antiapoptotic role [24]. Thus activation of Akt kinase induced by PTX (Figure 4) represents rather confusing result that have to be clarified by further study. Nevertheless, in the presence of PTX and VCR, *i.e.*, in condition when apoptosis progression in L1210/VCR cells was observed (Figure 2), the Akt kinase phosphorylation was declined to the levels only slightly higher than in control (Figure 4). A dose-dependent increase in the percentage of apoptotic cells after PTX treatment, mediated by a pathway that included cytochrome *c* release, caspase-3 activation and PARP cleavage, was also documented in HuT-78 cells [25]. These results suggest that the effect that PTX exerts on L1210/VCR cells is at least partially through PTX promotion of apoptosis. Mouse leukemia cells that developed MRD by overexpression of P-gp were found to also be cross-resistant to cisplatin, even though cisplatin is not a substrate of P-gp. These cells were found to be defective in cisplatin-induced initiation of apoptosis. This inability to enter apoptosis was associated with an imbalance between antiapoptotic and proapoptic proteins of the Bcl family [11]. The high expression levels of both P-gp and the antiapoptotic proto-oncogene Bcl-2 were reported to be associated with *in vitro* resistance to chemotherapeutic agents and a poor clinical outcome in cases of acute myeloid leukemia (AML) [26] and in cases of adults who have acute lymphoblastic leukemia [27].

Cancer cell growth and dissemination may be influenced by matrix metalloproteinases, which are secreted extracellularly. Matrix metalloproteinases are key extracellular matrix-degrading enzymes. A close association between the expression or activity of distinct matrix metalloproteinases and both tumor cell invasion and metastasis has been described [28]. Resistance of AML cells to anticancer drugs was described to be associated with increased activity and expression of MMP-2, which induced an enhancement in their invasivity [29]. This resistance of AML cells to anticancer drugs was also found to lead to a lack of drug-induced cell death, such as apoptosis.

An increased release of matrix metalloproteinases, which could be attenuated by PTX, was found in L1210/VCR cells, compared to parental L1210 cells. The doubling time of L1210/VCR cells, estimated to be 12 h, was shorter than the doubling time of 22 h observed for L1210 cells (data not shown). Thus, resistant cells could be considered as those with a higher proliferation rate. A direct connection between elevated activity of metalloproteinases in the extracellular matrix and cell proliferation was recently documented based on the release of extracellular matrix membrane-anchored growth factors [30]. This phenomenon may be related to a higher invasive ability of cells through upregulation of MMPs [16]. PTX was also found to inhibit proliferation of the melanoma B16F10 cells by inhibiting cell adhesion and secretion of matrix metalloproteinases [15].

Pentoxifylline is a substance which improves the sensitivity of several neoplastic cells to a large group of drugs that includes both substrates and nonsubstrates of P-gp [8,19,31]. In clinical conditions, PTX was described to prevent side-effects induced by irradiation during radiotherapy of breast cancer [32]. Our results indicate that this substance may also be useful in potentiating anticancer drugs’ effectiveness. However, this aspect has to be verified in clinical conditions.

## 3. Experimental Section

### 3.1. Materials

The primary antibodies used were the following: anti-Akt kinase, anti-MMP-2, anti-GAPDH (all from Santa Cruz Biotechnology Inc., Santa Cruz, CA, USA), anti-phospho-Akt kinase (reacts with Ser473-phosphorylated Akt kinase), anti-caspase-3, anti-cleaved caspase-3, anti-caspase-9, and anti-cleaved caspase-9 (all from Cell Signaling Technology Inc., Danvers, MA, USA). Pentoxifylline was obtained from Zentiva (formerly Slovakofarma, Hlohovec, Slovakia). Vincristine was supplied by Gedeon Richter (Budapest, Hungary).

### 3.2. Cell Culture and Exposure of Cells to Pentoxifylline and Vincristine

Parental sensitive mouse leukemia cell line L1210 and multidrug-resistant cell line L1210/VCR, which was obtained by long-term adaptation of sensitive cells to VCR, were used as experimental models (additional details characterizing this cell line are described elsewhere [11,33]). Both sensitive and resistant cells were grown under baseline conditions in RPMI-1640 medium supplemented with 4% fetal bovine serum in an incubator at 37 °C in a 5% CO_2_—95% air atmosphere. The L1210/VCR were cultured long term with addition of VCR (1.2 μmol/L) during each third passage. Sensitive L1210 and resistant L1210/VCR cells were exposed to 100 μmol/L PTX in the presence or absence of 1.2 μmol/L VCR for 24 h. These conditions were proved to be effective in P-gp transport activity and expression downregulation, in previous papers [9,19].

### 3.3. Preparation of Cellular Protein Fractions and Western Blot Analysis

After a cultivation period of 24 h, cells were centrifuged at 400 × g for 5 min, washed once with isotonic physiological solution and pelleted. To isolate the soluble protein fraction, pelleted cells were resuspended in ice-cold buffer containing 20 mmol/L Tris (pH 7.4), 1.0 mmol/L EGTA, 1.0 mmol/L DTT, 0.1 mmol/L sodium orthovanadate, and 0.5 mmol/L PMSF. After homogenization with a Teflon pestle homogenizer, the homogenate was centrifuged at 14,000 × g for 30 min at 4 °C. The supernatant was used for further analysis. Protein concentrations were determined according to the method of Bradford [34] using bovine serum albumin as a standard. Crude membrane fractions (used for P-gp determination) were isolated by processing pelleted cells according to manufacturer recommendations using the ProteoExtract Subcellular Proteome Extraction Kit (Calbiochem, CA, USA). Proteins in these samples were assessed using the Lowry protocol modified for membrane proteins by Peters *et al.* [35].

For Western blot analysis, 30 μg of each of the proteins was separated on SDS-polyacrylamide gels under reducing conditions [36]. Routinely, 10% gels were used with exception of P-gp detection where 8% gels were used. The proteins after electrophoretic separation were transferred onto nitrocellulose membranes, and after blocking of non-specific binding sites, the membranes were incubated overnight at 4 °C with the corresponding specific primary antibody [37]. The corresponding peroxidase-labeled anti-rabbit or anti-mouse immunoglobulins (Cell Signaling Technology Inc., Danvers, MA, USA) were used as secondary antibodies. Peroxidase reactions were detected by the enhanced chemiluminescence (ECL) system (GE Healthcare, Piscataway, NJ, USA, formerly Amersham Biosciences, UK).

### 3.4. Detection of Apoptotic Cells by FACS Analysis

Induction of apoptosis was detected by flow cytometry using the Annexin V-FITC Apoptosis detection kit (Sigma-Aldrich Corp., St. Louis, MO, USA). Sensitive or resistant cells in culture were preincubated in the absence or presence of VCR (1.2 μmol/L) and/or PTX (100 μmol/L) for 24 h. After this incubation, cells were washed twice with PBS and resuspended in 500 μL of binding buffer (10 mmol/L HEPES/NaOH, pH 7.5 containing 140 mmol/L NaCl and 2.5 mmol/L CaCl_2_) at a concentration of approximately 1 × 10^6^ cells/mL. Cell suspensions were added to plastic test tubes, and 5 μL of annexin V-FITC (50 μg/mL) and 10 μL of propidium iodide (100 μg/mL) were added to each cell suspension. The tubes were incubated at room temperature for 10 min, following which the fluorescence of the cells was determined using a Coulter Epics Altra flow cytometer (Beckman Coulter, Inc., Fullerton, CA, USA)

### 3.5. Effect of Pentoxifylline on the Release of Metalloproteinases from Sensitive L1210 and Drug-Resistant L1210/VCR Cells

Sensitive L1210 and resistant L1210/VCR cells cultured in RPMI-1640 medium supplemented with 4% fetal bovine serum were centrifuged at 400 × g for 4 min, washed twice with serum-free RPMI-1640 medium and resuspended in serum-free medium. Cells (5 × 10^5^ cells/mL) were then incubated in the absence of fetal bovine serum and exposed to 100 μmol/L PTX in the presence or absence of 1.2 μmol/L VCR. Conditioned medium was collected after 18 h, and cell-free supernatants were obtained by centrifugation. For each sample, 5 mL of cell-free supernatant was dialyzed against 20 mmol/L Tris-HCl buffer (pH 6.8), lyophilized and finally resuspended in 300 μL of 20 mmol/L Tris-HCl buffer (pH 6.8). To investigate the effects of PTX treatment on the secretion of matrix metalloproteinases from sensitive L1210 and resistant L1210/VCR cells, Western blot analysis with anti-MMP-2 antibody or gelatin zymography of concentrated conditioned medium samples was performed.

### 3.6. Gelatin Zymography

The samples of conditioned medium were mixed in a ratio of 1:1 with non-reducing Laemmli’s buffer and subjected to electrophoresis on 10% SDS-polyacrylamide gels co-polymerized with gelatin (final concentration of 2 g/L). After electrophoresis, gels were washed twice for 20 min each with 50 mmol/L Tris-HCl buffer (pH 7.4), containing 2.5% Triton X-100, at 25 °C. After washing, the gels were incubated overnight at 37 °C in substrate (developing) buffer containing 50 mmol/L Tris-HCl buffer (pH 7.4), 10 mmol/L CaCl_2_ and 1.25% Triton X-100. After this incubation, the gels were stained with 1% Coomassie Brilliant Blue G-250 dissolved in an aqueous solution containing 10% acetic acid and 40% methanol and then destained with an aqueous solution containing 10% acetic acid and 40% methanol. Gelatinolytic activities of MMP-2 and MMP-9 were detected as transparent bands against a dark blue background. Recombinant, active MMP-2 (Calbiochem, La Jolla, CA, USA) was used as a positive control.

### 3.7. Statistical Analysis

Data from three independent experiments were expressed as the mean ± SD. Statistical significance was assessed by an unpaired Student’s *t*-test.

## 4. Conclusions

Taken together, obtained results support our hypothesis that PTX may restore the sensitivity of neoplastic drug-resistant L1210/VCR cells to vincristine via the downregulation of P-gp protein expression. The PTX-induced restoration of L1210/VCR cells sensitivity to vincristine is associated with apoptosis induction through modulation of caspases activation. Further, we found that PTX influenced activation of Akt kinase. However, modulation of this kinase seems to be not directly involved in vincristine resistance reversal induced by PTX in L1210/VCR cells. PTX-induced improvement of vincristine cytotoxicity in L1210/VCR cells are also associated with modulation of extracellular release of MMPs.

All these aspects would be considered when elucidating the beneficial effects of PTX on neoplastic cells treated with anticancer drugs. Moreover, the obtained data bring new insights into the possible mechanisms of PTX action on cancer cells.
